# Food 4 Health - He Oranga Kai: Assessing the efficacy, acceptability and economic implications of *Lactobacillus rhamnosus* HN001 and β-glucan to improve glycated haemoglobin, metabolic health, and general well-being in adults with pre-diabetes: study protocol for a 2 × 2 factorial design, parallel group, placebo-controlled randomized controlled trial, with embedded qualitative study and economic analysis

**DOI:** 10.1186/s13063-019-3553-7

**Published:** 2019-07-29

**Authors:** Christine Barthow, Fiona Hood, Eileen McKinlay, Jo Hilder, Christine Cleghorn, Mark Huthwaite, Mark Weatherall, Amber Parry-Strong, Sue Pullon, Ben Gray, Kristin Wickens, Julian Crane, Jeremy Krebs

**Affiliations:** 1 Department of Medicine, University of Otago, PO Box 7343, Wellington South, Wellington, 6242 New Zealand; 2 Department of Primary Health Care & General Practice, University of Otago, PO Box 7343, Wellington South, Wellington, 6242 New Zealand; 3 Department of Public Health, University of Otago, PO Box 7343, Wellington South, Wellington, 6242 New Zealand; 4 Department of Psychological Medicine, University of Otago, PO Box 7343, Wellington South, Wellington, 6242 New Zealand; 5 Centre for Endocrine, Diabetes and Obesity Research (CEDOR), PO Box 7902, Wellington South, Wellington, New Zealand

**Keywords:** *Lactobacillus rhamnosus* HN001, Beta-glucan, Pre-diabetes, Probiotic, Prebiotic, DASS21, SF36, Acceptability, Economic analysis, Efficacy

## Abstract

**Background:**

The rates of pre-diabetes and type 2 diabetes mellitus are increasing worldwide, producing significant burdens for individuals, families, and healthcare systems. In New Zealand, type 2 diabetes mellitus and pre-diabetes disproportionally affect Māori, Pacific, and South Asian peoples. This research evaluates the efficacy, acceptability, and economic impact of a probiotic capsule and a prebiotic cereal intervention in adults with pre-diabetes on metabolic and mental health and well-being outcomes.

**Methods:**

Eligible adults (*n* = 152) aged 18–80 years with pre-diabetes (glycated haemoglobin 41–49 mmol/mol) will be enrolled in a 2 × 2 factorial design, randomised, parallel-group, placebo-controlled trial. Computer-generated block randomization will be performed independently. Interventions are capsulated *Lactobacillus rhamnosus* HN001 (6 × 10^9^ colony-forming units/day) (A) and cereal containing 4 g β-glucan (B), placebo capsules (O_1_), and calorie-matched control cereal (O_2_). Eligible participants will receive 6 months intervention in the following groups: AB, AO_1_, BO_2_, and O_1_O_2_. The primary outcome is glycated haemoglobin after 6 months. Follow-up at 9 months will assess the durability of response. Secondary outcomes are glycated haemoglobin after 3 and 9 months, fasting glucose, insulin resistance, blood pressure, body weight, body mass index, and blood lipid levels. General well-being and quality of life will be measured by the Short-Form Health Survey 36 and Depression Anxiety Stress Scale 21 at 6 and 9 months. Outcome assessors will be blind to capsule allocation. An accompanying qualitative study will include 24 face-to-face semistructured interviews with an ethnically balanced sample from the β-glucan arms at 2 months, participant focus groups at 6 months, and three health professional focus groups. These will explore how interventions are adopted, their acceptability, and elicit factors that may support the uptake of interventions. A simulation model of the pre-diabetic New Zealand population will be used to estimate the likely impact in quality-adjusted life years and health system costs of the interventions if rolled out in New Zealand.

**Discussion:**

This study will examine the efficacy of interventions in a population with pre-diabetes. Qualitative components provide rich description of views on the interventions. When combined with the economic analysis, the study will provide insights into how to translate the interventions into practice.

**Trial registration:**

Australian New Zealand Clinical Trials Registry, ACTRN12617000990325. Prospectively registered on 10 July 2017.

**Electronic supplementary material:**

The online version of this article (10.1186/s13063-019-3553-7) contains supplementary material, which is available to authorized users.

## Background

The prevalence of diabetes almost doubled worldwide between 1980 and 2016 [1]. This was predominantly driven by a rise in the rates of type 2 diabetes mellitus (T2DM) and pre-diabetes, which are associated with increasing rates of overweight or obesity [1]. In New Zealand, diabetes is one of the fastest growing long-term health conditions with an estimated increase of 7% per annum [2]. In addition, there are significant ethnic inequalities in the rates of diagnosed T2DM (2012/2013 data), with rates of 7% for Māori people, 13% for Pacific people, and 7% for Asian people, compared with 5.8% in the overall population [3], and pre-diabetes (2008/2009 data), with rates of 30% for Māori people and 30% for Pacific people, compared with 26% overall [4].

Pre-diabetes is an intermediate phase in the development of T2DM when glucose concentrations are between normal levels and those levels that define clinical diabetes [5]. Up to 70% of individuals with pre-diabetes will eventually develop T2DM [6]. Reversion to normal glucose levels, even if it is only transient, is associated with about half the risk for T2DM [7].

Diabetes is extremely costly to individuals, the healthcare system, and society as a whole [1, 2]. Healthcare costs are three times higher for an individual with diabetes compared with those for individuals without diabetes [2]. In addition, costs through loss of productivity exceed the healthcare costs [2].

Diabetes is associated with significant morbidities such as peripheral vascular disease, adult-onset blindness, renal failure, stroke, and cardiovascular disease (CVD) [8]. Depression is present at nearly double the normal rate in individuals with T2DM [9]. Lifestyle interventions, including dietary modification, exercise, and weight loss, are currently the main interventions used to reduce the risk of progression from pre-diabetes to T2DM [10]. However, these interventions are costly to implement and difficult to sustain. Additional effective measures, which are cost-effective and easy to implement on a broad scale, are required. To reduce health inequalities, such measures need to be acceptable and accessible to the groups at the highest risk of developing diabetes.

### Gut microbiota and health

Gut microbiota respond to environmental changes and moderate human physiology in multiple ways, which are not encoded for in the human genome [11, 12]. Dysbiosis (imbalance of microbial species in the body) has been associated with many health conditions including obesity, metabolic syndrome, non-alcoholic fatty liver disease, diabetes, and depression [13–15]. Therefore, the deliberate modulation of gut microbiota opens new possibilities in the search for ways to reduce the development of diabetes, CVD, and depression.

### Probiotics

Probiotics are defined by the World Health Organization as “live microorganisms which when administered in adequate amounts confer a health benefit to the consumer” [16]. Many different organisms are recognised as probiotics. Crucially, the health effects of probiotics are specific to the type, species, and strain of the probiotic used [17], and may be dose-dependent [18].

In a randomized controlled trial [19] we found that supplementation with the probiotic *Lactobacillus rhamnosus* HN001 (HN001) (6 × 10^9^ colony-forming units (cfu) daily) in pregnant women from 14 to 16 weeks gestation reduced the incidence of gestational diabetes, using the local New Zealand criteria for gestational diabetes, from 6.5% in the placebo group to 2.1% in the probiotic group (absolute risk difference 4.4%, *P* = 0.03) [20]. Notably this result was achieved without any other dietary or lifestyle modification. Post-natal depression scores and anxiety prevalence were also reduced in the probiotic arm of this study: the Edinburgh postnatal depression scale score for HN001, mean = 7.7 (standard deviation (SD) = 5.4) compared with 9.0 (SD 6.0) in the placebo group (*P* = 0.037); and anxiety present in 15.6% compared with 29.4% in the placebo group (adjusted *P* = 0.002) [21]. Our research extends this work to study the effect of the same probiotic in those with pre-diabetes, but with the addition of prebiotic/fibre intervention to optimize the potential health benefits.

### Prebiotics and fibre

Prebiotics are nondigestible high-fibre food components that are selectively fermented by particular members of the gut microbiota providing benefits to the health of the host [22]. Both soluble and insoluble fibre intake are important for prevention and treatment of T2DM, obesity, CVD, and hyperlipidaemia [23, 24].

Specifically, β-glucans are soluble polysaccharide prebiotics with a high molecular weight and are present in a range of noncereal and cereal foods, including oats and barley [25, 26]. The health properties of β-glucans are well documented [23, 26–31]. A 2018 meta-analysis of oat β-glucan (median dose 3.5 g/day) including data from 58 trials (*n* = 3974) found modest reductions in low-density lipoprotein (LDL; 0.19 mmol/l), non-high-density lipoprotein cholesterol (non HDL-C; 20 mmol/l), and apolipoprotein B (0.03 g/l) [32]. The European Food Safety Authority (EFSA) has endorsed claims that 4 g β-glucan significantly reduces blood glucose after meals, and 3 g/day decreases blood cholesterol [33]. However, to our knowledge there is little published evidence of the long-term effects of β-glucan and in particular whether these effects result in achievement and long-term maintenance of normal blood glucose concentrations, which is fundamental in decreasing the risk of developing T2DM.

Based on the above evidence of benefits from oat-derived β-glucan, in this research we will pair β-glucan with HN001. Furthermore, in vitro research indicates that β-glucan supports the growth of HN001 [34]. We contend that, in contrast to a probiotic-only intervention, this modification will most benefit metabolic and mental health outcomes.

In summary, diabetes is a significant and growing challenge worldwide with multiple social, economic, and health implications. Novel treatments are needed to help reduce the risk of pre-diabetes progressing to T2DM and in turn reduce the risk of the complications of diabetes. Building on our previous research, the Food 4 Health study: He Oranga Kai^1^ (F4H) utilises *L. rhamnosus* HN001 with the addition of the prebiotic β-glucan to quantitatively evaluate the health effects in adults with pre-diabetes. An in-depth understanding of the experiences of study participants in taking the study interventions, including their acceptability, and how these interventions are used by a range of ethnic groups including those most at risk of diabetes in New Zealand will be obtained through qualitative research methods. The study will seek health professional views on how to implement the intervention in practice. This, in combination with results from an economic analysis, will provide insights into how to translate the interventions into practice.

### Overarching aims

The overall goals of this work are: 1) to investigate the metabolic and other physical and psychological health effects of the single or combined use of daily HN001 probiotic and cereal enriched with 4 g β-glucan for 6 months in adults with pre-diabetes and the persistence of any health effects for a further 3 months after stopping the interventions; 2) to ascertain the acceptability of the study interventions, how they are adopted into participant’s lives, and develop a pathway to implementation; and 3) to establish the cost effectiveness of the intervention at the population level.

## Methods/design

### Randomized controlled trial design and hypotheses

This is a 2 × 2 factorial design, randomised, parallel-group, placebo-controlled trial in 152 adults with pre-diabetes designed to test the hypotheses that the administration of 6 months of the probiotic HN001 (6 × 10^9^ cfu) either alone or in combination with a cereal enriched with 4 g β-glucan to adults with pre-diabetes for 6 months will reduce glycated haemoglobin (HbA1c). The allocation ratio to groups will be 1:1:1:1. The design is to test the superiority of HN001 with or without 4 g β-glucan over placebo.

### Randomization and blinding

Randomization will be performed independently by Fonterra Co-Operative Ltd, using Microsoft Excel and a computer-generated random number list according to procedures explained by Kim and Shin [35]. Randomization will be performed in blocks of eight and not stratified; however, covariates will be collected and an important selection of these will be used in a sensitivity analysis.

The study is double-blinded for study capsules and only the study statistician will be unblinded after data analysis of the 6-month primary outcome. Outcome assessors and all participants will remain blinded to capsule allocation until all 9-month follow-up data are collected. Due to the obvious difference in cereals the study is not blinded for cereals; however, participants will not be informed of the full rationale for cereal choices, the full contents of cereal packages, or which cereal is considered the intervention and which is the control until after all participants have completed the study.

Fonterra Co-Operative Ltd will share the study cereal allocation codes with the study centre, while capsule allocations will remain double-blinded until data are analysed.

Staff will allocate the next study number in consecutive sequence to those eligible at the commencement of the enrolment visit. The type of cereal allocated will only be revealed to the participant at the time the relevant intervention is dispensed after baseline measures are collected.

### Setting and participants

Study participants will be predominantly urban-dwelling adults, recruited from the community in the greater Wellington region of New Zealand.

Eligible individuals will be English speaking adults aged 18–80 years who are capable of giving informed consent, who have pre-diabetes, defined as per New Zealand criteria as a screening HbA1c 41–49 mmol/mol (5.9–6.6%) [36]. There is potential for a change in HbA1c between screening and enrolment. To minimize the possibility that those with screening HbA1cs in the lower range will have regressed to the normal range by enrolment, the following criteria will apply. Those with screening HbA1c in the range 41–44 mmol/mol will be enrolled within 4 months of the screening test date, and those with HbA1c in the range 45–49 mmol/mol will enrolled within 1 year of the screening test date. For analysis, only the HbA1c at baseline visit and subsequent time points will be included. Regardless of the baseline HbA1c value, all participant data will be included in the analysis.

In addition, participants will be excluded if any of the following apply:

- Previous bariatric surgery, or pre-existing T1DM or T2DM. Where a previous diagnosis of T2DM is uncertain, this will be defined as ever having had two consecutive HbA1c results ≥ 50 mmol/mol that are at least 3 months apart
- Abnormal thyroid function (thyroid-stimulating hormone < 0.3 mU/l or ≥ 4.5 mU/l in the last 18 months); those requiring stable doses of thyroxine for treated hypothyroidism will be eligible for the study
- Current significant renal disease (estimated glomerular filtration rate < 30 ml/min/1.72m^2^)
- Serious immune dysfunction (for example HIV/AIDS, immune deficiency diseases)
- Current pregnancy or breastfeeding, or planning to conceive during the study
- Unstable body weight (active weight loss/gain > 5 kg in prior 3 months), or weight > 200 kg
- Structural heart disease (known valve abnormality or replacement, history of endocarditis) or other vascular implant, apart from cardiac stents or intracranial coils
- Gastrointestinal disorder that alters the digestion and absorption of nutrients (e.g. ulcerative colitis, Crohn’s disease, an ileostomy or colostomy)
- Medication use; current use of immune suppressant medications, medications that modify blood sugar levels, or anticipated regular use of such medications (e.g. frequent use of oral or injected steroids), long-term use of systemic antibiotics
- Use fibre/prebiotic/β-glucan/probiotic supplements and is unable or unwilling to cease using these for the study duration
- Allergy/intolerances; coeliac disease, intolerance to oats, milk, barley or corn or severe allergy to traces of other allergens that may be present in the study interventions
- Does not agree to refrain from donating blood for 3 months prior to each study visit
- Is participating in, or has recently participated in, another research study involving an intervention which may alter outcomes of interest to this study, or lives with another participant who is already enrolled in the F4H study
- Does not have a general practitioner/primary healthcare provider or does not agree to study communicating clinical results to the general practitioner if needed
- Any other condition or situation which, in the view of investigators, would affect the compliance or safety of the individual taking part

### Interventions

#### Study capsules

Active study capsules will contain *L. rhamnosus* HN001 (6 × 10^9^ cfu) and 140 mg corn-derived maltodextrin. *L. rhamnosus* powder will be produced as described by Wickens et al. [37]. Placebo capsules will be identical in appearance and contain 150 mg (corn-derived) maltodextrin. Grain Processing Corp., Oregon, USA, manufacture the maltodextrin, and Salkat New Zealand Ltd, Auckland, will supply it to Fonterra Co-operative Group Ltd. Alaron Products Ltd, Nelson, New Zealand, will encapsulate both powders. An external registered laboratory will undertake safety and quality testing to pharmaceutical standards and compliance with the Therapeutic Goods Act. Fonterra Co-Operative Ltd will supply the capsules in sealed opaque bottles containing 105 capsules.

Study capsules will be maintained in cool storage (4 °C) to preserve probiotic viability. Participants will be instructed to store capsules in their home fridge, and to contact the study centre if they are accidentally left at room temperature for more than 2 days, or left in the hot sun for 30 min or more; under these circumstances, replacement capsules will be issued. Participants will be instructed to take one capsule a day and avoid taking the capsule 10 min before or after eating or drinking hot substances.

Throughout the study, Fonterra Co-Operative Ltd will test capsules returned from the field to ensure probiotic viability is maintained at a minimum of 6 × 10^9^ cfu.

#### Study cereals

Cereals were chosen as the delivery method for the β-glucan prebiotic intervention to enable an adequate dose to be administered in the intervention cereal. In addition, cereals can be administered as a food in a range of formats to increase the variety of options for participants.

The active cereal contains 40 g Uncle Toby’s Flemings Rolled Oats (manufactured by Nestle Australia) and 8 g of OatWell® 28XF oatbran (supplied by DSM Nutritional Products Ltd, Switzerland) or 8 g SWEOAT Bran BG28 XF (the same product; supplied by Swedish Oat Fiber AB, Sweden) (www.sweoat.com). Total β-glucan dose is 4 g. Oatwell 28/SWEOAT Bran BG28 XF is a heat-treated extra-fine oatbran containing 28% high-viscosity-solubility and high-molecular-weight (> 2000 kDa) β-glucan.

The control cereal contains 35 g cornflakes (manufactured by Sanitarium Health and Wellbeing, Auckland, New Zealand) and 8 g non-dairy creamer (C35; manufactured by Shantou City Chenghai District Wenhui Food Co. Ltd, China).

Cornflakes and creamer were chosen as the control cereal as they are expected to have minimal prebiotic effect. Having a control cereal and powder enhances the study design by enabling some masking of which cereal contains the active interventions. Active and control ereals are calorie matched. See Additional file 1 for comparison of cereal nutritional values.

HealthPak Ltd, Penrose, Auckland, New Zealand, will pack the study cereals and supply them in single-serve daily portions. To ensure precise dosing, each individual daily serving contains two opaque packs, one containing the course-grained cereal (oats/cornflakes) and a smaller sachet containing the powder (β-glucan or non-dairy creamer) The two sachets which together constitute a daily serving of cereal are enclosed together in a clear outer package.

Study participants take the full contents of one cereal pack daily for 6 months. Cereals will be used as a replacement for some aspect of the participant’s normal diet, may be taken at any time of the day, and may be taken at a different time to the study capsule if desired. Standard instructions include avoiding or minimizing the addition of sugar, honey, or golden or maple syrup to the cereals to no more than a teaspoon. A recipe card containing serving suggestions will be supplied to each participant. These suggestions were designed through consultation with the ethnic groups known to be at higher risk of diabetes in New Zealand. Recipes are designed to encourage compliance with the study cereals by providing a variety of ways to take the cereals (e.g. hot, cold, or as a smoothie), appeal to differing ethnic food preferences, and actively encourage the use of whole foods to flavour the cereals.

#### Concomitant care

To ensure all participants have access to a similar baseline knowledge of pre-diabetes, all participants will be given a standard information pamphlet about pre-diabetes at the enrolment visit. Study participants will be advised to use the study interventions daily (as described above), and apart from this to continue with their current routines. All participants will be given a record of their anthropometric measures at each study visit. Physical activity levels and dietary intake will be measured by the Stanford Leisure-Time Activity Categorical Item questionnaire (L-Cat 2.2) [38] and 3-day diet diary at every study time point. These data will be used in the analysis to control for the effects of changes in activity and diet.

Throughout the study, all participants will continue with their usual healthcare provided by their general practitioner. Any change to care such as commencing medications which may impact on study outcomes will be collected and adjusted for during the analysis.

#### Adverse effects and modification to study interventions

In previous HN001 studies we have found the probiotic to be safe and well tolerated [20, 39].

All components of the cereal interventions are marketed for human consumption and are therefore not a safety risk. While some individuals require gradual increases when introducing a higher level of fibre into their diet, consumption up to 10 g β-glucan daily is generally tolerated [30]. β-glucans undergo fermentation in the colon, and participants may experience flatulence, bloating, or alteration in bowel habits [30]. Any gastrointestinal effects of interventions will be systematically assessed in questionnaires included in the 3-, 6-, and 9-month follow-up visits.

If any participant reports gastrointestinal symptoms which may relate to the study interventions that are causing discomfort, pain, or impacting on the participant’s ability to take the study interventions, and are not severe enough to require medical review, the following procedure will be followed:

1. Discontinue both capsule and cereal interventions until symptoms settle. In instances where participants clearly relate symptoms to the cereal then they stop the cereal only and continue with the capsule.
2. If symptoms resolve, then they recommence capsules only and continue for a few days to check that no symptoms return.
3. If capsules are tolerated, they begin to reintroduce the cereal. Start the cereal reintroduction by mixing powder with cereal and taking half of the total dose only. Then, after a few days and if tolerated well, gradually increase the dose to the full amount.
4. If at any point symptoms return, they reduce the dose to the level that they can tolerate.
5. If required, to improve tolerability the daily cereal dose may be taken in part doses spread over the day.
6. It is very unlikely that participants would not be able to tolerate either of the interventions; however, should this occur the participant will be encouraged to continue in the study with interventions at levels they can tolerate, and to ensure outcome data are collected for an intention-to treat analysis. Compliance with the interventions will be collected (see below).
7. If symptoms do not resolve on discontinuation of study capsule and cereals, then advise participant to see their usual healthcare provider for further advice and investigation; withhold study interventions until a diagnosis is made and the symptoms are resolved. Then consider reintroduction as per 2–5 above.

#### Adherence to study interventions

Multiple strategies will be used to encourage and monitor adherence to the study interventions. Clear verbal and written instructions will be provided at the enrolment and 3-month visit. Discussion will include assessment of lifestyle patterns and how best to remember to take study capsules, and how to integrate cereals into the individuals’ pattern of eating. Practical aids will be offered, including recipe sheets, a blender for making smoothies, and a reusable 1-week medication dispenser to assist with recalling if the daily capsule has been taken. Two weeks after commencing the study all participants will be contacted to assess how they are managing with taking the interventions; further individualized coaching will be provided if required. In addition, similar discussions will occur at any other time a participant indicates this is needed, and routinely to all participants at the 3-month visit to encourage ongoing adherence.

All unused study capsules and cereals will be returned and counted.

### Outcome measures

The primary outcome is HbA1c after 6 months. HbA1c is chosen as it is currently the most commonly used diagnostic test for pre-diabetes and T2DM in New Zealand [40], and therefore has the most direct relevance to clinical practice.

Secondary outcomes include HbA1c after 3 months and 9 months, which is 3 months after the cessation of the interventions to check if there are persistent effects. Other secondary outcomes include fasting glucose, insulin resistance measured by homeostatic model assessment, fasting lipid profiles, mean systolic and diastolic blood pressure, and anthropometric measures (waist size, body weight, and body mass index (BMI)) at 3 and 6 months. Psychological outcomes include symptoms of depression, anxiety, and stress measured on the shortened version of the Depression Anxiety Stress Scale (DASS 21) [41] and health-related quality of life measured by the short-form health survey version 2 for New Zealand/Australia (SF-36) [42] after 6 and 9 months only. See Table 1 for a summary of outcome measures, and Table 2 for a summary of questionnaire variables. Study questionnaires were pre-tested during the study set up.

**Table 1:** Summary of outcome measures

Outcomes	Time point	Measurement
Primary outcome		
HbA1c	At 6 months	Measured from whole blood from K_2_EDTA tube on a Cobas c 311, Roche Diagnostics, Auckland, New Zealand
Secondary outcomes		
HbA1c	At 3 and 9 months	Measured from whole blood from K_2_EDTA tube on a Cobas c 311, Roche Diagnostics, Auckland, New Zealand
Fasting plasma glucose	At 3, 6, and 9 months	Measured from plasma from fluoride/oxalate tube on a Cobas c 311, Roche Diagnostics, Auckland, New Zealand
Fasting insulin		Measured from plasma from K_2_EDTA by Human/Canine/Porcine Insulin DuoSet ELISA R&D Systems Inc.
HOMA-IR		Calculated as HOMA = (glucose (mmol/l) x insulin (pmol/l))/22.5 [43]
Fasting lipid profiles		Measured from fasting serum from SST II Advance Tube as total cholesterol, low-density lipoprotein cholesterol, high-density lipoprotein cholesterol, and triglycerides. Measured on a Cobas c 311, Roche Diagnostics, Auckland, New Zealand
Blood pressure	At 3 and 6 months	Measured as mean systolic and mean diastolic blood pressure using standard calibrated electronic sphygmomanometer and protocol*
Weight		Measured in kg using standard calibrated electronic equipment and protocol*
Waist circumference		Measured in cm using standard equipment and protocol*
Body mass index		Calculated as weight (kg)/height (m)^2^
Depression, anxiety, and stress scores	At 6 and 9 months	Measured using DASS 21. The DASS is a set of three self-report scales designed to measure states of depression, anxiety and stress. It has validated psychometric properties, with good test–retest properties in both clinical and community populations, and is robust across a range of demographic variables [44–48]
Physical and emotional well-being		Measured using SF-36. The SF-36 is a self-completed questionnaire, relating to physical and emotional health over the past 4 weeks. It is a well-validated scale, with good psychometric properties, widely used as a health outcome measure [42, 49–51]. Raw data will be uploaded and analysed on the ‘QualityMetric Health Outcomes TM scoring software 5.1. Permission to use granted on 13 December 2017
Other outcomes		
Adverse events	At 3, 6, and 9 months	Hospitalizations
		Gastrointestinal symptoms and bowel function
Compliance	At 3 and 6 months	Calculated based on return of unused cereal and capsules

*See Additional file 2 for equipment and protocols for anthropometric and blood pressure measures

*DASS 21* Shortened version of the Depression Anxiety Stress Scale, *HbA1c* glycated haemoglobin, *HOMA-IR* homeostatic model assessment of insulin resistance, *SF-36* Short-form health survey version 2 for New Zealand/Australia

**Table 2:** Summary of questionnaire variables

Questions	Time point collected	Details	Source
Demographics	Baseline	Name, address, date of birth, ethnicity, income, educational qualifications	Questions based on Statistics New Zealand Census questions 2013, available from http://archive.stats.govt.nz/Census/2013-census/info-about-the-census/forms-guidenotes.aspx.
Diabetes and cardiovascular disease risk factors	Baseline	History of first-degree relative with type 2 diabetes mellitus. Personal history of gestational diabetes, or polycystic ovarian disease (females only), hypertension, dyslipidaemia, myocardial infarction, stoke, mental illness, ethnicity, smoking	Questions based on Ministry of Health 2016 New Zealand Health Survey Adult Questionnaire (Year 6) 1 July 2016–30 June 2017 with some specific additions. New Zealand Health survey available from https://www.health.govt.nz/publication/content-guide-2016-17-new-zealand-health-survey
History of mental health disorders	Baseline and at 6 and 9 months if newly diagnosed since previously collected	History of anxiety, depression, or other mental health illness; current treatments used	Questions based on Ministry of Health 2016 New Zealand Health Survey Adult Questionnaire (Year 6) 1 July 2016–30 June 2017 with some specific additions. New Zealand Health survey available from https://www.health.govt.nz/publication/content-guide-2016-17-new-zealand-health-survey
Recent major stressful life events	Baseline and at 6 and 9 months	History of stressful life events occurring in the preceding 12 months at baseline, and since last recorded at subsequent time points	The Life Stress scale is adapted from the Pregnancy Risk Assessment Monitoring System (Centers for Disease Control and Prevention, 2009), and is a 13-item measure included to address the occurrence of recent stressful experiences
Medications	All	Name, dose, frequency, route of all prescribed medications (except topical), and specific over-the-counter medications	Developed by researchers coded according to World Health Organization Anatomical Therapeutic Chemical Classification System with Defined Daily Doses [52]
Supplement use	All	Confined to probiotics, prebiotic/fibre, fish oils, and plant sterols supplements; name, frequency of use, types of probiotics and prebiotics contained in relevant supplements	Developed by researchers
Background use of foods which may impact outcomes	All	Confined to foods containing probiotics (yogurt and other fermented foods), oats/corn, artificial sweeteners, foods enriched with plant sterols; frequency of use in past month (at baseline) or since previous visit (all other time points)	Developed by researchers
Professional lifestyle modification or mental health advice	All	Has participant received professional advice about lifestyle interventions or mental health (in previous 12 months at baseline, and since last collected at other time points)	Created by researchers based on questions from Ministry of Health 2016 New Zealand Health Survey Adult Questionnaire (Year 6) 1 July 2016–30 June 2017 with some specific additions. New Zealand Health survey available from https://www.health.govt.nz/publication/content-guide-2016-17-new-zealand-health-survey
3-day food diary	All	All foods and drinks including quantities and methods of preparation over 3 days	Data entered into Foodworks 9 (Xyris Software Australia Pty Ltd)
Physical activity measure	All	Six-point descriptive categorical scale used to assess levels of activity, and in relation to recommended activity guidelines. Categories broadly match activity intensity based on metabolic equivalents.	Stanford Leisure-Time activity categorical Item (L-Cat 2.2) [38]
Smoking	All	Classification as non-smoker, ex-smoker, or current smoker and intensity of current smoking	Developed by researchers
Alcohol and recreational drug use	Baseline and at 6 and 9 months	Frequency of alcohol use and number standard drinks taken of a typical day. Use of substances for recreational or non-medicinal purposes to get high. Identifies substances used and frequency of use	Questions based on Ministry of Health 2016 New Zealand Health Survey Adult Questionnaire (Year 6) 1 July 2016–30 June 2017 with some specific additions. New Zealand Health survey available from https://www.health.govt.nz/publication/content-guide-2016-17-new-zealand-health-survey
Gastrointestinal symptoms	At 3, 6 and 9 months	Changes in frequency of gastrointestinal symptoms including nausea/vomiting, stomach aches, cramps, or pain, bloating or swollen stomach/abdomen, and bowel motions. Evaluation of impact of any changes in bowel pattern.	Developed by researchers
Hospitalizations	At 3, 6 and 9 months	Hospitalizations since previous visit (excluding elective surgery and outpatient appointments)	Developed by researchers
Use of reduced dose of cereals	At 3 and 6 months	Yes/no question to determine if participant usually takes half or less of the daily dose of corn/oats, and powder sachet	Developed by researchers

### Sample size

There are few published studies reporting HbA1c change following lifestyle or pharmaceutical intervention in pre-diabetes. For example, a trial in those with pre-diabetes by Defronzo et al. [53] on a drug intervention using pioglitazone reported a point estimate of the reduction in HbA1c of 0.24% (2.6 mmol/mol) relative to placebo. In T2DM a clinically important difference with drug interventions is 5 mmol/mol. Past research reports an SD for HbA1c in similar clinical samples to those with pre-diabetes of between 4 and 6 mmol/mol [54]. Based on the larger of these SDs, a sample size of 32 in each main effects arm has 90% power to detect a 5 mmol/mol reduction with a type I error rate of 5%. Accounting for 25% drop-out, a total sample size of 88 is needed (22 in each factorial combination). The power to detect differences is likely to be improved using baseline HbA1c as a continuous covariate. However, an uncertainty for this study is whether the minimal clinically important difference for reduction of HbA1c in the setting of pre-diabetes should be anticipated to be closer to that seen in the pioglitazone study (increasing the required sample size), and that the SD for the HbA1c is likely to be smaller in pre-diabetes than in T2DM (decreasing the required sample size). For 80% power to detect a difference of 2.6 mmol/mol with an SD of 4 and 25% drop-out, a total recruitment of 92 is needed, and for 90% power *n* = 136. For a difference of 3.8 mmol/mol, half-way between the two values, and the larger SD of 6, a total sample size of 152 participants with 38 in each factorial combination will be recruited for this study.

### Analysis plan

All primary analyses will be based on intention-to-treat principles with all individuals analysed in the group to which they were randomized. Demographic and baseline characteristics will be summarised using descriptive statistics. The primary outcome is HbA1c at 6 months. The primary analysis for the main outcome variable will be analysis of covariance (ANCOVA) with the baseline measurement of HbA1c as a continuous covariate, and the randomized treatments as main effects, and their interaction as categorical explanatory variables. Adjusted analyses will be conducted to include potential baseline confounding variables (BMI, level of exercise and total daily energy intake), and whether medications that could affect glucose metabolism were used after randomization. The effect of change in weight, change in exercise, and change in total daily energy intake between baseline and 6 months on HbA1c will also be explored using ANCOVA. A linear mixed model using all of the three HbA1c measurements and an appropriate random effects structure to account for correlation between repeated measurements, and using HbA1c at baseline as a continuous covariate, will be used as a sensitivity analysis for the effect of the randomized treatments. Subgroup analysis will use interaction terms (with treatment) for whether treatment effects vary by ethnicity, sex, or socioeconomic status.

Secondary outcome variables will be analysed in a similar way, with the most important outcomes for each being the 6-month time point. All outcome variables will be analysed with their baseline value as a continuous covariate. Subsequent sensitivity analyses will be guided by the outcome of this, with the introduction of relevant baseline covariates for each outcome variable. Factors which may affect mental health and well-being outcomes such as alcohol, recreational drug use, and life stressors will be described and considered in the analysis. Additional file 3 contains tables for baseline characteristics and results.

### Study sequence

**Fig. 1:**
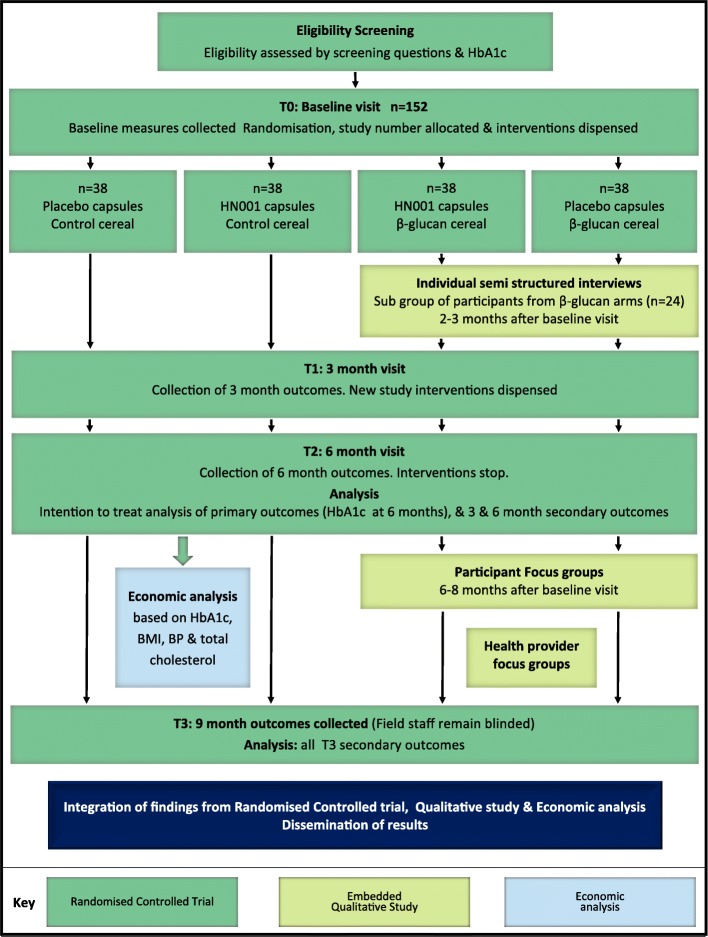
Flow chart of study. BMI body mass index, BP blood pressure, HbA1c glycated haemoglobin, HN001 *Lactobacillus rhamnosus* HN001

**Fig. 2:**
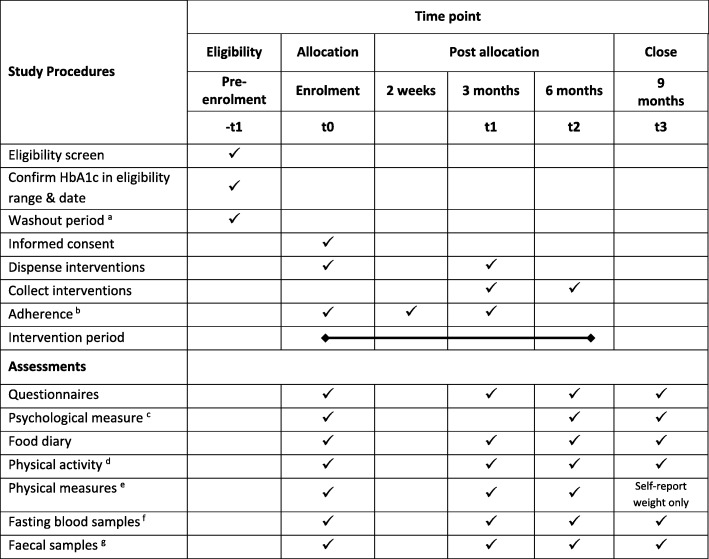
Study timeline and procedures. ^a^ Washout period of 1 month if subject has used probiotic supplements, systemic antibiotics, or short-term systemic steroids prior to enrolment. ^b^ Discussions/contacts to maintain adherence and study participation. ^c^ Shortened version of the Depression Anxiety Stress Scale (DASS 21) and Short-form health survey version 2 for New Zealand/Australia (SF-36). ^d^ Stanford Leisure-Time activity categorical Item (L-Cat 2.2). ^e^ Weight, height (and body mass index), blood pressure. ^f^ Glycated haemoglobin (HbA1c), fasting glucose, insulin, and lipids and additional bloods bio-banked for mechanistic studies. ^g^ Faecal samples to enable examination of possible mechanistic effects of study interventions

Figures 1 and 2 represent the study sequence and timelines for procedures, respectively. Additional file 4 contains the Standard Protocol Items: Recommendations for Interventional Trials (SPIRIT) Checklist.

### Recruitment

Multiple recruitment strategies will be used to recruit participants to the study. Working with local primary health organizations, individuals with pre-diabetes will be invited by letter to join the study. In addition, the general public will be notified about the study through study posters, study information sessions, relevant email lists, and other hardcopy and online media.

All those expressing an interest in the study will be assessed for eligibility by a screening questionnaire and measurement of HbA1c.

### Information and consent

Potential participants will be provided with trial information through study videos available on a study website, written information and consent forms, and detailed discussions with field staff prior to screening and study enrolment. Information and consent forms will cover participation in the F4H study, and a separate information and consent form for the collection of additional biological samples (faecal samples and blood) to be bio-banked for later analysis.

Field research staff will obtain formal written informed consent at the commencement of the enrolment visit. Participants will attend four morning study visits in a fasted state. See Fig. 2 for the study timeline.

### Data and sample collection

Study data will be collected and managed using Research Electronic Data Capture (REDCap) tools hosted at the University of Otago, Dunedin, New Zealand. REDCap is a secure, web-based application designed to support data capture for research studies, providing: 1) an intuitive interface for validated data entry; 2) audit trails for tracking data manipulation and export procedures; 3) automated export procedures for seamless data downloads to common statistical packages; and 4) procedures for importing data from external sources. Field staff will complete the majority of data input; however, participants will self-complete the more sensitive questions online. These include the DASS 21, SF36, income, smoking, alcohol, drug use, mental health history, physical exercise, and bowel symptom questions. In addition, the 9-month data collection will not require a face-to-face visit with the field staff. Apart from those who are unable to complete questionnaires electronically and require a telephone interview, participants will complete all 9-month questionnaires online.

Field staff will use inbuilt REDcap data queries to check the completeness of all data and inbuilt range checks will be used to prevent data entry errors. Any changes to data will be recorded in the REDcap data log, and records of any data, which is checked or amended, will be completed using the REDcap database’s data query system.

#### Staff training

To ensure that high-quality and consistent data are collected, all field staff will be trained in the administration of study questionnaires and assessments of physical measures according to standard study protocols.

Field staff will instruct the participant on the completion of the SF36 using standard recommended procedures [42]. Anthropometric and blood pressure data will be collected using clearly defined protocols, standardized and calibrated equipment, and with duplicate readings and range checks to ensure data reliability. See Additional file 2 for equipment and protocols for anthropometric and blood pressure measures.

Staff trained in phlebotomy procedures will collect fasting blood according to a standardized protocol. After centrifuging, aliquots of serum or plasma will be stored at −80 °C until processed. Analysis will be performed in batches containing all of an individual’s samples to minimize measurement error due to batch variability. Those consenting to the collection of faecal samples will collect these samples in the 2–3 days prior to each study visit immediately after completing the 3-day food diary. See Additional file 5 for details on the blood and faecal sample collection and quality-control protocols.

#### Protocol deviations

Any participants who temporarily or permanently cease taking study interventions will be encouraged to remain in the study and data will be collected as per protocol. If such participants are unwilling to complete all further assessments, the first priority will be collection of primary outcome data (HbA1c at 6 months and related questions), followed by all other 6-month data. If participants withdraw completely, data collected to the time of their withdrawal will be included in the intention-to-treat analysis, and reasons for withdrawal will be reported.

#### Access to data

Only fieldworkers will have direct access to the study database. Participants will be allocated a unique study code, and data will be de-identified prior to export from the database or analysis. Data will be stored securely for at least 10 years and a possible indefinite period following this in accordance with the consent procedures for this study.

### Data monitoring

Due to the low-risk nature of the project, internal safety and data monitoring will occur by the named investigators. There will be no interim analysis of results and trial-stopping guidelines are considered unnecessary.

To monitor the study, reports of any participants ceasing the interventions and reasons for this will be presented at monthly meetings, and further investigations made if any concerns are identified. If for any reason unbinding to study capsule allocation is required, this will be done by a non-fieldworker contacting the randomization code holder at Fonterra Co-Operative Ltd.

Analysis of HbA1c, lipids, and mental well-being outcomes will occur after all participants have completed the 6-month intervention to inform the qualitative study and economic analysis (see below).

### Qualitative study

The accompanying qualitative study aims to explore whether participants in the β-glucan-based cereal arms find interventions acceptable or not in order to enhance the robustness of the study findings and inform translation to real-life settings [55–58]. The qualitative study will include: 1) approximately 24 qualitative semistructured interviews with participants from the two β-glucan cereal arms of the randomized controlled study, and the same participants will join one of four focus groups; and 2) three focus groups each with about eight to ten health professionals to discuss how they might translate the findings into long-term use in real-life clinical settings. It is intended that the qualitative data be collected in tandem with quantitative methods and purposefully integrated into study findings [59, 60].

#### Participants

A purposive sample of around 24 participants, six each from Māori*,* Pacific, South Indian and other ethnic groups enrolled in the β-glucan cereal arms, will be invited to join the qualitative study. We will use diversity sampling to include a gender, age, education, and socioeconomic mix. Participants will be asked to take part in an individual interview at 2 months and join one of four focus groups at around 6 months following completion of the intervention.

A purposive sample of health professionals will be invited to join the study as part of three focus groups. Diversity sampling will include a range of health promoters, community health workers, dieticians, health navigators, general practitioners, nurses, and pharmacists.

#### Data collection

A combination of individual interviews and focus groups will be used. Individual interviews allow one-to-one engagement, whereas focus groups prompt for additional information/revelations through social interaction not likely elicited individually [61]. The face-to-face audio-recorded individual interviews will follow a semistructured interview schedule. Questions will include how the interventions have been used, factors which may influence adherence, social and cultural beliefs about food, health, probiotics, pre-diabetes, diabetes, food, and health literacy, as well as potential for behaviour change and self-management. The focus group interview schedule will be informed by the data from the individual interviews and will further explore the acceptability of the interventions, real-world experience, differences between Māori, Pacific, South Indian and other ethnicities, and forecast how the interventions could be used in the long term. Focus groups with health professionals will explore their views on the acceptability of the intervention, barriers or facilitators to implementation, and draw on their experiences working with people with pre-diabetes or diabetes in general.

#### Analysis

The interviews will be transcribed verbatim. Transcripts will be imported into Nvivo 11 and a researcher (JH) will code in two ways: first, according to the content framework of questions [62, 63]; and second, according to meaning units and themes [64, 65]. The coding will then be shared with the research team (EM, BG, and SP), and consensus will be obtained through discussion and the testing of examples to ensure reliability.

### Economic analysis

The aim of the economic analysis is to model the potential cost-effectiveness of the intervention if it were implemented in a wider New Zealand context. The change in HbA1c, BMI, blood pressure, and serum total cholesterol observed at 6-month follow-up in the randomized controlled trial will be used in the model as estimates of the effectiveness of the intervention. These changes in risk factors will be modelled through to a change in incidence of the relevant diseases and then onto changes in quality-adjusted life years (QALYs), costs or cost savings to the health system (in 2011 New Zealand dollars; NZ$) and incremental cost-effectiveness ratios which show the health system cost per QALY gained. A health system perspective will be used, and benefits and costs will be modelled using a lifetime horizon. Outputs will be discounted at 3%.

The intervention will be modelled using a multi-state life-table (MSLT) model populated with rich New Zealand health data. An existing MSLT model, which is parameterised for the whole of the New Zealand population [66, 67], will be re-parameterised to the New Zealand population with pre-diabetes using data from routinely collected administrative health data (Healthtracker and the Integrated Data Infrastructure) and the Adult Nutrition Survey 2008/2009. In order to estimate the difference in QALYs and health system costs between the model’s intervention and business-as-usual (BAU) comparator, the entire New Zealand population alive in 2011 will be simulated out until death.

The BAU model uses projected all-cause mortality and morbidity rates by sex, age, and ethnicity (Māori and non-Māori). The model includes 14 diseases related to the included risk factors where proportions of the population simultaneously reside: coronary heart disease (CHD), stroke, T2DM, osteoarthritis, and multiple cancers (i.e. endometrial, kidney, liver, oesophageal, pancreatic, thyroid, colorectal, breast, ovarian, and gallbladder). The proportion of the New Zealand population in each disease life-table is a function of the disease incidence, case fatality, and remission (the latter in cancers only). Morbidity will be quantified (separately by sex, age, and ethnic group) for each disease using the years of life lived with disability (YLDs) from the New Zealand Burden of Disease Study divided by the population count to give prevalent YLDs. Disability weights from the Global Burden of Disease Study 2010 will be used to estimate the health status valuation of these YLDs [68].

Relative risks for the association between the relevant risk factors (HbA1c, BMI, blood pressure, and serum total cholesterol) and disease incidence will be sourced from the Global Burden of Disease study [69] or recent meta-analyses. These relative risks will be combined with the changes in risk factors through population impact fractions [70] to give the proportionate change in future disease incidence. The model will also capture the change in CHD and stroke incidence from the changing incidence of diabetes.

Time lags from the change in intervention-related risk factors to change in disease incidence is allowed for by using the average risk factor change over a previous window of time of 0 to 5 years for CVD, diabetes, and osteoarthritis, and 10 to 30 years for cancers. Probabilistic uncertainty about the boundaries (5, 10, and 30 years) will also be specified.

The proportionate change in disease incidence changes the ‘inflow’ to each of the multiple parallel disease sheets in the model. Each 5-year age cohort alive in 2011 is modelled out until their death in one ‘overall’ master life-table. The cohort is rewarded with health (i.e. QALYs) and health system costs in each annual cycle. This ‘overall’ master life-table has annual mortality, morbidity and costs that are mathematically linked to the multiple parallel disease life-tables. As the inflow (i.e. disease incidence) to a parallel disease life-table reduces (e.g. falling diabetes incidence), this reduces the morbidity, mortality, and costs of disease.

Health system costs (sex- and age-specific) will be calculated in 2011 NZ$ using individually linked data for publicly funded (and some privately funded) health events occurring in 2006–2010, including hospitalizations, inpatient procedures, outpatients, pharmaceuticals, laboratories, and expected primary care usage. Building on an existing framework [71] for calculating the timing of health system costs, the whole cohort will be assigned a sex- and age-specific annual health system cost of a citizen without an intervention-related disease and not in the last 6 months of their life. Additional disease-specific excess costs will be assigned to people: 1) in the first year of an intervention-related disease diagnosis; 2) in the last 6 months of life if dying of the given disease; and 3) otherwise prevalent cases of each disease. Costs will be modelled over the lifetime of the cohort, including costs both related and unrelated to the modelled diseases (meaning increased longevity due to the intervention will contribute to increased health system costs for some cohort members). How the intervention will be scaled up to the national level will be conceptualised, including estimating the cost of the intervention (see Cleghorn et al. [66] for an example of our methods). These estimates will be used in the model as estimates of the proportion of the total pre-diabetic population in New Zealand that will receive the intervention.

The excel model will be run 2000+ times using Monte Carlo simulation through an Ersatz add-in which samples from the uncertainty interval around each input parameter. This allows the calculation of QALYs and costs with the appropriate amount of uncertainty. Results from this programme of research will be disseminated widely. See Additional file 6.

## Discussion

Type 2 diabetes is one of the most important long-term health conditions in the world, with major impact on lives of individuals and a burden to health systems. Pre-diabetes offers an opportunity to intervene to prevent the progression to T2DM. Although diet and lifestyle interventions have been shown to be efficacious, widespread effectiveness of these has been challenging. Therefore, any additional effective intervention which is acceptable and accessible will be very valuable. In addition, depression and anxiety are also important and increasing long-term conditions, which similarly require additional effective interventions. The role of the gut microbiome in health is rapidly becoming more apparent, and interventions that can modify the microbiome are showing promise in multiple health conditions. Both probiotics and prebiotics have the potential to do this and form the basis of the intervention in this study.

This paper describes the protocol of a mixed-methods study including a randomized controlled trial of the probiotic HN001 compared with placebo in a 2 × 2 factorial design with an active prebiotic cereal and a control cereal. The study also includes a qualitative study to enrich the findings of the quantitative study and inform the translation of the findings into real-world practice. In addition, an economic analysis will provide the cost-effectiveness of the intervention to inform funders. If effective, this intervention would be a simple, additional treatment option for people with pre-diabetes that could be promoted within primary care, and also at a public health level.

## Trial registration

This trial was prospectively registered on 10 July 2017 on the Australian New Zealand Clinical Trials Registry, registration ACTRN12617000990325. See https://www.anzctr.org.au/ACTRN12617000990325.aspx. The Universal Trial Number of this trial is U1111-1195-7561.

## Trial status

This paper is based on protocol version 2.1 dated 28 September 2018. Recruitment began in February 2018, and approximate date of completion is April 2019. Any significant protocol modifications will be notified to the ethics committee and updated on the Australian New Zealand Clinical trial registry.

## Additional files

Additional file 1: Food 4 Health - He Oranga Kai: Comparison of cereal nutritional values. (PDF 622 kb)

Additional file 2: Food 4 Health - He Oranga Kai: Equipment and protocols for anthropometric and blood pressure measures. (PDF 620 kb)

Additional file 3: Food 4 Health - He Oranga Kai:Tables for baseline characteristics and results. (PDF 798 kb)

Additional file 4: Food 4 Health - He Oranga Kai: Standard Protocol Items - Recommendations for Interventional Trials (SPIRIT) Checklist. (PDF 243 kb)

Additional file 5: Food 4 Health - He Oranga Kai: Blood and faecal sample collection and quality control protocols. (PDF 705 kb)

Additional file 6: Food 4 Health - He Oranga Kai: Dissemination strategy. (PDF 612 kb)

## Data Availability

Data sharing is not applicable to this article as no datasets are reported. Availability of datasets generated in the study will be included in papers reporting study outcomes. Access to the full protocol and model consent forms may be available from the author upon reasonable request.

## Abbreviations

BAU: Business-as-usual
BMI: Body mass index
cfu: Colony-forming units
CHD: Coronary heart disease
CVD: Cardiovascular disease
DASS 21: Shortened version of the Depression Anxiety Stress Scale
F4H: Food 4 Health study: He Oranga Kai
HbA1c: Glycated haemoglobin
HDL-C: High-density lipoprotein cholesterol
HN001: *Lactobacillus rhamnosus* HN001
LDL: Low-density lipoprotein
MSLT: Multi-state life-table
QALY: Quality-adjusted life year
REDCap: Research Electronic Data Capture
SD: Standard deviation
SF-36: Short-form health survey version 2 for New Zealand/Australia
T2DM: Type 2 diabetes mellitus
YLDs: Years of life lived with disability

## Footnotes

1 He Oranga Kai translated from Māori means Food for Health.

## References

[1] World Health Organization. Global report on diabetes. Geneva; World Health Organization; 2016.

[2] Ministry of Health (2015). Living well with diabetes: a plan for people at high risk of or living with diabetes 2015–2020.

[3] Ministry of Health. New Zealand Health Survey annual update of key findings 2012/13. Wellington: Ministry of Health; 2013.

[4] Coppell KJ, Mann JI, Williams SM, Jo E, Drury PL, Miller J (2013). Prevalence of diagnosed and undiagnosed diabetes and prediabetes in New Zealand: findings from the 2008/09 Adult Nutrition Survey. N Z Med J.

[5] Grundy SM (2012). Pre-diabetes, metabolic syndrome, and cardiovascular risk. J Am Coll Cardiol.

[6] Tabak AG, Herder C, Rathmann W, Brunner EJ, Kivimaki M (2012). Prediabetes: a high-risk state for diabetes development. Lancet.

[7] Perreault L, Pan Q, Mather KJ, Watson KE, Hamman RF, Kahn SE (2012). Effect of regression from prediabetes to normal glucose regulation on long-term reduction in diabetes risk: results from the Diabetes Prevention Program Outcomes study. Lancet.

[8] Dagogo-Jack S (2002). Preventing diabetes-related morbidity and mortality in the primary care setting. J Natl Med Assoc.

[9] Roy T, Lloyd CE (2012). Epidemiology of depression and diabetes: a systematic review. J Affect Disord.

[10] Diabetes Prevention Program Research Group (2002). Reduction in the incidence of type 2 diabetes with lifestyle intervention or metformin. N Engl J Med.

[11] Kovatcheva-Datchary P, Arora T (2013). Nutrition, the gut microbiome and the metabolic syndrome. Best Pract Res Clin Gastroenterol.

[12] Shen J, Obin MS, Zhao L (2013). The gut microbiota, obesity and insulin resistance. Mol Asp Med.

[13] Bäckhed F, Fraser CM, Ringel Y, Sanders ME, Sartor RB, Sherman PM (2012). Defining a healthy human gut microbiome: current concepts, future directions, and clinical applications. Cell Host Microbe.

[14] Han J, Lin H (2014). Intestinal microbiota and type 2 diabetes: from mechanism insights to therapeutic perspective. World J Gastroenterol.

[15] Wang H, Lee IS, Braun C, Enck P (2016). Effect of probiotics on central nervous system functions in animals and humans—a systematic review. J Neurogastroenterol Motil.

[16] Food and Agriculture Organization and World Health Organization. Health and nutritional properties of probiotics in food including powder milk with live lactic acid bacteria. 2001. http://www.fao.org/tempref/docrep/fao/meeting/009/y6398e.pdf. Accessed 16 July 2019.

[17] Hampe CS, Roth CL (2017). Probiotic strains and mechanistic insights for the treatment of type 2 diabetes. Endocrine.

[18] Ouwehand AC (2017). A review of dose-responses of probiotics in human studies. Benef Microbes.

[19] Barthow C, Wickens K, Stanley T, Mitchell EA, Maude R, Abels P (2016). The Probiotics in Pregnancy Study (PiP study): rationale and design of a double-blind randomised controlled trial to improve maternal health during pregnancy and prevent infant eczema and allergy. BMC Pregnancy Childbirth.

[20] Wickens Kristin L., Barthow Christine A., Murphy Rinki, Abels Peter R., Maude Robyn M., Stone Peter R., Mitchell Edwin A., Stanley Thorsten V., Purdie Gordon L., Kang Janice M., Hood Fiona E., Rowden Judy L., Barnes Phillipa K., Fitzharris Penny F., Crane Julian (2017). Early pregnancy probiotic supplementation with Lactobacillus rhamnosus HN001 may reduce the prevalence of gestational diabetes mellitus: a randomised controlled trial. British Journal of Nutrition.

[21] Slykerman RF, Hood F, Wickens K, Thompson JMD, Barthow C, Murphy R (2017). Effect of Lactobacillus rhamnosus HN001 in pregnancy on postpartum symptoms of depression and anxiety: a randomised double-blind placebo-controlled trial. EBioMedicine.

[22] Kootte RS, Vrieze A, Holleman F, Dallinga-Thie GM, Zoetendal EG, de Vos WM (2012). The therapeutic potential of manipulating gut microbiota in obesity and type 2 diabetes mellitus. Diabetes Obes Metab.

[23] El Khoury D, Cuda C, Luhovyy BL, Anderson GH (2012). Beta glucan: health benefits in obesity and metabolic syndrome. J Nutr Metab.

[24] Daou C, Zhang H (2012). Oat beta-glucan: its role in health promotion and prevention of diseases. Compr Rev Food Sci Food Saf.

[25] Smith CE, Tucker KL (2011). Health benefits of cereal fibre: a review of clinical trials. Nutr Res Rev.

[26] Tosh SM (2013). Review of human studies investigating the post-prandial blood-glucose lowering ability of oat and barley food products. Eur J Clin Nutr.

[27] Ahmad A, Anjum FM, Zahoor T, Nawaz H, Dilshad SMR (2012). Beta glucan: a valuable functional ingredient in foods. Crit Rev Food Sci Nutr.

[28] Cloetens L, Ulmius M, Johansson-Persson A, Åkesson B, Önning G (2012). Role of dietary beta-glucans in the prevention of the metabolic syndrome. Nutr Rev.

[29] Tiwari U, Cummins E (2011). Meta-analysis of the effect of beta-glucan intake on blood cholesterol and glucose levels. Nutrition.

[30] Lam KL, Chi-Keung Cheung P (2013). Non-digestible long chain beta-glucans as novel prebiotics. Bioact Carbohydr Diet Fibre.

[31] Jayachandran M, Chen J, Sum S, Chung M, Xu B (2018). A critical review on the impacts of β-glucans on gut microbiota and human health. J Nutr Biochem.

[32] Ho HVT, Sievenpiper JL, Zurbau A, Blanco Mejia S, Jovanovski E, Au-Yeung F (2016). The effect of oat β-glucan on LDL-cholesterol, non-HDL-cholesterol and apoB for CVD risk reduction: a systematic review and meta-analysis of randomised-controlled trials. Br J Nutr.

[33] EFSA Panel on Dietetic Products Nutrition and Allergies (NDA) (2011). Scientific opinion on the substantiation of health claims related to beta-glucans from oats and barley and maintenance of normal blood LDL-cholesterol concentrations (ID 1236, 1299), increase in satiety leading to a reduction in energy intake (ID 851, 852), reduction of post-prandial glycaemic responses (ID 821, 824), and “digestive function” (ID 850) pursuant to Article 13(1) of Regulation (EC) No 1924/2006. EFSA J.

[34] Sims IM, Ryan JLJ, Kim SH (2014). Invitro fermentation of prebiotic oligosaccharides by Bifidobacterium lactis HN019 and Lactobacillus spp. Anaerobe.

[35] Kim J, Shin W (2014). How to do random allocation (randomization). Clin Orthop Surg.

[36] New Zealand Guidelines Group (2011). Guidance on the management of type 2 diabetes 2011.

[37] Wickens K, Black PN, Stanley TV, Mitchell E, Fitzharris P, Tannock GW (2008). A differential effect of 2 probiotics in the prevention of eczema and atopy: a double-blind, randomized, placebo-controlled trial. J Allergy Clin Immunol.

[38] Kiernan M, Schoffman DE, Lee K, Brown SD, Fair JM, Perri MG (2013). The Stanford Leisure-Time Activity Categorical Item (L-Cat): a single categorical item sensitive to physical activity changes in overweight/obese women. Int J Obes.

[39] Dekker JW, Wickens K, Black PN, Stanley T, Mitchell EA, Fitzharris P (2009). Safety aspects of probiotic bacterial strains Lactobacillus rhamnosus HN001 and Bifidobacterium animalis subsp. lactis HN019 in human infants aged 0-2 years. Intern Dairy J.

[40] Ministry of Health (2018). Cardiovascular disease risk assessment and management for primary care.

[41] Lovibond SH, Lovibond PF (1995). Manual for the Depression Anxiety Stress Scales.

[42] Maruish ME (2011). User’s manual for the SF-36v2 health survey.

[43] Matthews DR, Hosker JR, Rudenski AS, Naylor BA, Treacher DF, Turner RC (1985). Homeostasis model assessment: insulin resistance and fl-cell function from fasting plasma glucose and insulin concentrations in man. Diabetologia.

[44] Lovibond PF, Lovibond SH (1995). The structure of negative emotional states: comparison of the Depression Anxiety Stress Scales (DASS) with the Beck Depression and Anxiety Inventories. Behav Res Ther.

[45] Crawford JR, Henry JD (2003). The Depression Anxiety Stress Scales (DASS): normative data and latent structure in a large non-clinical sample. Br J Clin Psychol.

[46] Ronk FR, Korman JR, Hooke GR, Page AC (2013). Assessing clinical significance of treatment outcomes using the DASS-21. Psychol Assess.

[47] Henry JD, Crawford JR (2005). The short-form version of the Depression Anxiety Stress Scales (DASS-21): construct validity and normative data in a large non-clinical sample. Br J Clin Psychol.

[48] Page AC, Hooke GR, Morrison D (2007). Psychometric properties of the Depression Anxiety Stress Scales (DASS) in clinical samples. Br J Clin Psychol.

[49] Ware JE (2000). SF-36 health survey update. Spine (Phila Pa 1976).

[50] Brazier J, Harper R, Jones N, O’Cathain A, Thomas K, Usherwood T (1992). Validating the SF-36 health survey questionnaire: new outcome measure for primary care. BMJ.

[51] Chittleborough CR, Baldock KL, Taylor AW, Phillips PJ (2006). Health status assessed by the SF-36 along the diabetes continuum in an Australian population. Qual Life Res.

[52] WHO Collaborating Centre for Drug Statistics Methodology (2016). Guidelines for ATC classification and DDD assignment.

[53] DeFronzo RA, Tripathy D, Schwenke DC, Banerji M, Bray GA, Buchanan TA (2011). Pioglitazone for diabetes prevention in impaired glucose tolerance. N Engl J Med.

[54] Parker AR, Byham-Gray L, Denmark R, Winkle PJ (2014). The effect of medical nutrition therapy by a registered dietitian nutritionist in patients with prediabetes participating in a randomized controlled clinical research trial. J Acad Nutr Diet.

[55] Moffatt S, White M, Mackintosh J, Howel D (2006). Using quantitative and qualitative data in health services research—what happens when mixed method findings conflict? [ISRCTN61522618]. BMC Health Serv Res.

[56] Protheroe J, Bower P, Chew-Graham C (2007). The use of mixed methodology in evaluating complex interventions: identifying patient factors that moderate the effects of a decision aid. Fam Pract.

[57] Palinkas Lawrence A., Horwitz Sarah M., Green Carla A., Wisdom Jennifer P., Duan Naihua, Hoagwood Kimberly (2013). Purposeful Sampling for Qualitative Data Collection and Analysis in Mixed Method Implementation Research. Administration and Policy in Mental Health and Mental Health Services Research.

[58] Midgley N, Ansaldo F, Target M (2014). The meaningful assessment of therapy outcomes: incorporating a qualitative study into a randomized controlled trial evaluating the treatment of adolescent depression. Psychotherapy.

[59] Lewin S, Glenton C, Oxman AD (2009). Use of qualitative methods alongside randomised controlled trials of complex healthcare interventions: methodological study. BMJ.

[60] O’Cathain A, Murphy E, Nicholl J (2010). Three techniques for integrating data in mixed methods studies. BMJ.

[61] Ziebland S, Coulter A, Calabrese JD, Locock L. Understanding and using health experiences: improving patient care. Oxford: Oxford Scholarship Online; 2013.

[62] Graneheim UH, Lundman B (2004). Qualitative content analysis in nursing research: concepts, procedures and measures to achieve trustworthiness. Nurse Educ Today.

[63] Gale N, Health G, Cameron E, Rashid S, Redwood S (2013). Using the framework method for the analysis of qualitative data in multi-disciplinary health research. BMC Med Res Methodol.

[64] Braun VCV (2006). Using thematic analysis in psychology. Qual Res Psychol.

[65] Braun V, Clarke V (2014). What can “thematic analysis” offer health and wellbeing researchers?. Int J Qual Stud Health Well-being.

[66] Cleghorn Christine, Wilson Nick, Nair Nisha, Kvizhinadze Giorgi, Nghiem Nhung, McLeod Melissa, Blakely Tony (2019). Health Benefits and Cost-Effectiveness From Promoting Smartphone Apps for Weight Loss: Multistate Life Table Modeling. JMIR mHealth and uHealth.

[67] Cleghorn C, Blakely T, et al. Technical report for BODE^3^ intervention and DIET MSLT models, Version 1. Burden of Disease Epidemiology, Equity and Cost-Effectiveness Programme. Technical Report no. 16. Wellington: Department of Public Health, University of Otago, Wellington; 2017.

[68] Salomon J, Vos T, Hogan D, Gagnon M, Naghavi M, Mokdad A, Begum N, Shah R, Karyana MKS (2012). Common values in assessing health outcomes from disease and injury: disability weights measurement study for the Global Burden of Disease Study 2010. Lancet.

[69] Forouzanfar MH, Alexander L, Anderson HR (2015). Global, regional, and national comparative risk assessment of 79 behavioural, environmental and occupational, and metabolic risks or clusters of risks in 188 countries, 1990–2013: a systematic analysis for the Global Burden of Disease Study 2013. Lancet.

[70] Barendregt JJ, Veerman JL (2010). Categorical versus continuous risk factors and the calculation of potential impact fractions. J Epidemiol Community Health.

[71] van Baal P, Feenstra T, Polder J, Hoogenveen RBW (2011). Economic evaluation and the postponement of health care costs. Health Econ.

