# Supplementary material for: Food 4 Health - He Oranga Kai: Assessing the efficacy, acceptability and economic implications of Lactobacillus rhamnosus HN001 and β-glucan to improve glycated haemoglobin, metabolic health, and general well-being in adults with pre-diabetes: study protocol for a 2 × 2 factorial design, parallel group, placebo-controlled randomized controlled trial, with embedded qualitative study and economic analysis
Source: Trials. 2019 Jul 29;20:464. doi: 10.1186/s13063-019-3553-7 (PMC6664750; doi:10.1186/s13063-019-3553-7)
Supplement: Supplementary file 1 — Food 4 Health - He Oranga Kai: Comparison of cereal nutritional values. (PDF 622 kb) [file 13063_2019_3553_MOESM1_ESM.pdf]

Additional file 1

Food 4 Health – He Oranga Kai: Comparison of cereal nutritional values

	**Active Cereal**					**Control Cereal**				
	Oats		Oatwell 28%XF or SWEOAT BRAN BG28 XF		**Total Per dose Prebiotic cereal**	Cornflakes		Creamer		**Total Per dose control cereal**
	per 100g	Per 40 g	per 100g	Per 8g		per 100g	Per 35 g	per 100g	Per 8g	
**Energy (kj)**	1600.0	640.0	1160.0	92.8	**732.8**	1550.0	542.5	2315.0	185.2	**727.7**
**Protein (g)**	12.8	5.1	23.0	1.8	**7.0**	7.4	2.5	2.0	0.2	**2.8**
**Fat, total**	9.2	3.7	5.0	0.4	**4.1**	0.3	0.1	35.0	2.8	**2.9**
**Saturated**	1.7	0.7	1.0	0.1	**0.8**	0.1	0.0	34.4	2.8	**2.8**
**Carbohydrate**	56.7	22.7	9.0	0.7	**23.4**	82.1	28.7	58.0	4.6	**33.4**
**Sugars**	1.0	0.4		0.0	**0.4**	7.7	2.9	14.0	1.1	**3.8**
**Dietary fibre**	9.2	3.7	52.0	4.2	**7.8**	2.7	0.9			**0.9**
**Soluble**	4.5	1.8	28.0	2.2	**4.0**					
**Insoluble**	5.7	2.3	24.0	1.9	**4.2**					
**Beta glucan**	4.5	1.8	28.0	2.2	**4.0**					
**Sodium (mg)**	6.0	2.4		0.0	**2.4**	550.0	199.5			**199.5**
