# Supplementary material for: Food 4 Health - He Oranga Kai: Assessing the efficacy, acceptability and economic implications of Lactobacillus rhamnosus HN001 and β-glucan to improve glycated haemoglobin, metabolic health, and general well-being in adults with pre-diabetes: study protocol for a 2 × 2 factorial design, parallel group, placebo-controlled randomized controlled trial, with embedded qualitative study and economic analysis
Source: Trials. 2019 Jul 29;20:464. doi: 10.1186/s13063-019-3553-7 (PMC6664750; doi:10.1186/s13063-019-3553-7)
Supplement: Supplementary file 2 — Food 4 Health - He Oranga Kai: Equipment and protocols for anthropometric and blood pressure measures. (PDF 620 kb) [file 13063_2019_3553_MOESM2_ESM.pdf]

Additional File 2

Food 4 Health – He Oranga Kai: Equipment and protocols for Anthropometric and Blood pressure measures

Variable	Equipment	Protocol
Height (cms)	Seca 213 Mobile stadiometer for measuring height. Manufactured by Hammer Steindamm 9 – 25, 22089 Hamburg, Germany. www.seca.com	Measured without shoes, and hair down.<br>Stand the participant on the base plate with weight distributed evenly on both feet, heels together and at the back of the stadiometer base plate, toes pointing outward at 60 degree angle.<br>Participant's buttocks, upper back and head touching or in line with stadiometer upright.<br>Arms at side and head positioned in frankfort plane. Participant breathes in, stretches to fullest height and the headboard is brought down to measure.<br>Measures are repeated until two measures differ by no more than 0.5cm.<br>Protocol based on NZ Health survey(1) Protocol for collecting Height, weight and waist measurements in NZ health surveys(2) & Centres for Disease Control and Prevention. Anthropometry procedures manual (3).
Weight (kgs)	Tanita TIHD351 Person Scale Manufactured by Tanita Corporation, 2625 South Clearbrook Drive, Arlington Heights, Illinois 60005, USA www.tanita.com	Measured on a hard surface.<br>Participant removes shoes and heavy outer clothing<br>Scales are activated and zero<br>Participant positioned on centre of scales with weight evenly distributed.<br>Take measure when the scaled have stabilised on reading<br>Weight recorded to nearest 0.1kg<br>Repeat measures until two measures differ by no more than 0.5kg<br>Protocol based on NZ Health survey(1)
Waist circumference (cms)	Seca 201 measuring tape Manufactured by Hammer Steindamm 9 – 25, 22089 Hamburg, Germany. www.seca.coma	Measure directly against skin, or if participant refuses measure over only a minimal layer of clothing<br>Participant stands with feet together and weight evenly balanced<br>Position tape firmly midway between the inferior margin of the last rib and crest of the ilium in a horizontal plane.<br>Measure to nearest 0.1cm at end of normal expiration<br>Reposition tape and repeat measures until 2 measures differ by no more than 1cm

		Protocol based on NZ Health survey(1) Protocol for collecting Height, weight and waist measurements in NZ health surveys(2) & Centres for Disease Control and Prevention. Anthropometry procedures manual (3).
Blood Pressure	Omron HBP1300 Professional blood pressure monitor Manufactured by OMRON HEALTHCARE Co., Ltd	Participant is seated and at rest for 5 minutes before reading is taken<br>BP measure is taken on left arm.<br>Arm positioned at heart level and resting on firm surface at the same level as heart<br>Apply correct size of BP cuff firmly to bare arm 2.5 cm above the bend in the elbow, and ensure that the cuff index arrow is over the brachial artery<br>Instruct participant to remain still and quiet. Take 3 readings 1 minute apart. The first reading is discarded, 2nd and 3rd readings are recorded.<br>Protocol based on NZ health survey (1) and Ormron Blood pressure monitor instructions

References

1. New Zealand Ministry of Health. New Zealand Health Survey Adult Questionnaire (Year 6) 1 July 2016 – 30 June 2017 CAPI Version In field July 2016 [Internet]. 2016 [cited 2018 Nov 24]. Available from: https://www.health.govt.nz/publication/content-guide-2016-17-new-zealand-health-survey
2. Public Health Intelligence. Protocol for Collecting Height , Weight and Waist Measurements in New Zealand Health Monitor (NZHM) Surveys. 2008;1–7.
3. Centres for Disease Control and Prevention. Anthropometry procedures manual. Natl Heal Nutr examinatory Surv. 2007;(January):102.
