# Supplementary material for: Food 4 Health - He Oranga Kai: Assessing the efficacy, acceptability and economic implications of Lactobacillus rhamnosus HN001 and β-glucan to improve glycated haemoglobin, metabolic health, and general well-being in adults with pre-diabetes: study protocol for a 2 × 2 factorial design, parallel group, placebo-controlled randomized controlled trial, with embedded qualitative study and economic analysis
Source: Trials. 2019 Jul 29;20:464. doi: 10.1186/s13063-019-3553-7 (PMC6664750; doi:10.1186/s13063-019-3553-7)
Supplement: Supplementary file 3 — Food 4 Health - He Oranga Kai:Tables for baseline characteristics and results. (PDF 798 kb) [file 13063_2019_3553_MOESM3_ESM.pdf]

Additional File 3

Food 4 Health - He Orangi Kai: Tables for baseline characteristics and results

Table 1: Baseline demographic details and comorbid conditions

	HN001	Beta-glucan	HN001+beta-glucan	Placebo
**Demographic characteristics**	N=	N=	N=	N=
Age (in years) (Mean, SD)	Mean (SD)	Mean (SD)	Mean (SD)	Mean (SD)
Gender (male)	n (%)	n (%)	n (%)	n (%)
Ethnicity[a]				
Maori ethnicity	n (%)	n (%)	n (%)	n (%)
Pacific ethnicity	n (%)	n (%)	n (%)	n (%)
Indian/South Asian[b]	n (%)	n (%)	n (%)	n (%)
Other Asian ethnicity	n (%)	n (%)	n (%)	n (%)
European/Pakeha ethnicity	n (%)	n (%)	n (%)	n (%)
Other ethnicity	n (%)	n (%)	n (%)	n (%)
Household income ($NZ)				
0-49,000	n (%)	n (%)	n (%)	n (%)
50-99,000	n (%)	n (%)	n (%)	n (%)
100-149,000	n (%)	n (%)	n (%)	n (%)
150,000 +	n (%)	n (%)	n (%)	n (%)
**History of co-morbid conditions**				
1st degree relative with T2 diabetes	n (%)	n (%)	n (%)	n (%)
Gestational diabetes (if previous pregnancy>20weeks)	n (%)	n (%)	n (%)	n (%)
Polycystic ovarian syndrome (if female)	n (%)	n (%)	n (%)	n (%)
Heart disease[c]	n (%)	n (%)	n (%)	n (%)
Hypertension	n (%)	n (%)	n (%)	n (%)
Dislipidemia	n (%)	n (%)	n (%)	n (%)
Stroke	n (%)	n (%)	n (%)	n (%)
Depression[d]	n (%)	n (%)	n (%)	n (%)
Anxiety	n (%)	n (%)	n (%)	n (%)
Other mental health illness	n (%)	n (%)	n (%)	n (%)

[a]Ethnicity is defined as single ethnicity with order of priority as Maori, Pacific, Indian/South Asian, South East Asian,

[b]Indian/South Asian includes Indian, Fijian Indian, Bangladeshi, Pakistani, Afghani, Bhutanese, Nepalese, Sri Lankan and Maldivian.

[c]Heart disease is defined as having a history of angina, heart failure or heart attack.

[d]Depression is defined participant ever told by a health professional that they were depressed or needed anti-depressant medication

[e]Anxiety is defined as participant ever told by a health professional that they were anxious or needed treatment for anxiety

[f]Other mental health condition is defined as other condition mental health condition for which participant is currently receiving treatment

Table 2: Baseline distribution of physical activity level, diet, smoking, alcohol consumption, Recreational drug use, medication or supplement use. *(potential confounders or effect modifiers)*

	HN001	Beta-glucan	HN001+beta-glucan	Placebo
**Physical activity level**				
Inactive	n (%)	n (%)	n (%)	n (%)
Light (1-2 times a week)	n (%)	n (%)	n (%)	n (%)
Moderate (3+ times a week)	n (%)	n (%)	n (%)	n (%)
Vigorous (3+ times a week)	n (%)	n (%)	n (%)	n (%)
**Diet (once a week or more)**				
Yoghurt	n (%)	n (%)	n (%)	n (%)
Fermented foods	n (%)	n (%)	n (%)	n (%)
Artificial sweeteners	n (%)	n (%)	n (%)	n (%)
Oats	n (%)	n (%)	n (%)	n (%)
Total calorie intake[a]	Mean (SD)	Mean (SD)	Mean (SD)	Mean (SD)
**Smoking**				
Current smoker	n (%)	n (%)	n (%)	n (%)
**Prescribed and OTT Medications**				
Antibiotics in last 6 months	n (%)	n (%)	n (%)	n (%)
Oral or injected steroids	n (%)	n (%)	n (%)	n (%)
Proton pump inhibitors/Antacids	n (%)	n (%)	n (%)	n (%)
Antihypertensives/diuretics	n (%)	n (%)	n (%)	n (%)
Lipid lowering medications	n (%)	n (%)	n (%)	n (%)
Bowel medications	n (%)	n (%)	n (%)	n (%)
Antidepressant/anxiolytic				
**Supplements containing**				
Fibre/Prebiotic	n (%)	n (%)	n (%)	n (%)
Fish oil	n (%)	n (%)	n (%)	n (%)

[a]Estimated from 3-day food diary

Table 2 will be repeated at 3, 6 and 9 months and include use of non-study probiotics.

Table 3: Baseline values of participant outcome measures

Outcomes	HN001	Beta-glucan	HN001+beta-glucan	Placebo
**Metabolic outcome measures**	N=	N=	N=	N=
HbA1c (mmol/mol) (%)	Mean (SD)	Mean (SD)	Mean (SD)	Mean (SD)
Fasting serum glucose (mmol/L)	Mean (SD)	Mean (SD)	Mean (SD)	Mean (SD)
Insulin resistance (HOMA-IR)	Mean (SD)	Mean (SD)	Mean (SD)	Mean (SD)
Fasting Lipids (mmol/L)				
Total Cholesterol	Mean (SD)	Mean (SD)	Mean (SD)	Mean (SD)
LDL	Mean (SD)	Mean (SD)	Mean (SD)	Mean (SD)
HDL	Mean (SD)	Mean (SD)	Mean (SD)	Mean (SD)
Triglycerides	Mean (SD)	Mean (SD)	Mean (SD)	Mean (SD)
Anthropometry				
Weight (kg)	Mean (SD)	Mean (SD)	Mean (SD)	Mean (SD)
BMI (weight (kg)/height (m^2)	Mean (SD)	Mean (SD)	Mean (SD)	Mean (SD)
Waist circumference (cm)	Mean (SD)	Mean (SD)	Mean (SD)	Mean (SD)
Blood pressure (mmHg)				
Systolic	Mean (SD)	Mean (SD)	Mean (SD)	Mean (SD)
Diastolic	Mean (SD)	Mean (SD)	Mean (SD)	Mean (SD)
**Mental Health outcome measures**	N=	N=	N=	N=
DASS Total score	Mean (SD)	Mean (SD)	Mean (SD)	Mean (SD)
DASS Depression subscale	Mean (SD)	Mean (SD)	Mean (SD)	Mean (SD)
DASS Anxiety subscale	Mean (SD)	Mean (SD)	Mean (SD)	Mean (SD)
DASS Stress subscale	Mean (SD)	Mean (SD)	Mean (SD)	Mean (SD)
SF 36	Mean (SD)	Mean (SD)	Mean (SD)	Mean (SD)
Physical Functioning	Mean (SD)	Mean (SD)	Mean (SD)	Mean (SD)
Role Physical	Mean (SD)	Mean (SD)	Mean (SD)	Mean (SD)
Bodily Pain	Mean (SD)	Mean (SD)	Mean (SD)	Mean (SD)
General Health	Mean (SD)	Mean (SD)	Mean (SD)	Mean (SD)
Vitality	Mean (SD)	Mean (SD)	Mean (SD)	Mean (SD)
Social Functioning	Mean (SD)	Mean (SD)	Mean (SD)	Mean (SD)
Role Emotional	Mean (SD)	Mean (SD)	Mean (SD)	Mean (SD)
Mental Health	Mean (SD)	Mean (SD)	Mean (SD)	Mean (SD)
Physical Component Summary PCS	Mean (SD)	Mean (SD)	Mean (SD)	Mean (SD)
Mental Component Summary (MCS)	Mean (SD)	Mean (SD)	Mean (SD)	Mean (SD)

Table 4 The adjusted[a] effects of HN001 and B-glucan and their interaction on the primary and secondary outcome variables.

	HN001				B-glucan				
	Yes	No			Yes	No			
	Mean (SD)	Mean (SD)	Mean diff (95% CI)	P=	Mean (SD)	Mean (SD)	Mean diff (95% CI)	P=	P for interaction
**Primary outcome**									
HbA1c(mmol/mol) at 6 mths (%)	xx.x (xx)	xx.x (xx)	xx.x (95% CI)	P=	xx.x (xx)	xx.x (xx)	xx.x (95% CI)	P=	P=
**Secondary outcomes**									
HbA1c(mmol/mol) at 3 mths (%)	xx.x (xx)	xx.x (xx)	xx.x (95% CI)	P=	xx.x (xx)	xx.x (xx)	xx.x (95% CI)	P=	P=
HbA1c(mmol/mol) at 9 mths (%)	xx.x (xx)	xx.x (xx)	xx.x (95% CI)	P=	xx.x (xx)	xx.x (xx)	xx.x (95% CI)	P=	P=
**Other secondary metabolic outcomes**									
Fasting glucose (mmol/L) at 3,6,9 mths	xx.x (xx)	xx.x (xx)	xx.x (95% CI)	P=	xx.x (xx)	xx.x (xx)	xx.x (95% CI)	P=	P=
Insulin resistance (HOMA-IR) at 3,6,9 mths	xx.x (xx)	xx.x (xx)	xx.x (95% CI)	P=	xx.x (xx)	xx.x (xx)	xx.x (95% CI)	P=	P=
Fasting Lipids (mmol/L) at 3,6,9 mths									
Cholesterol mmol/L	xx.x (xx)	xx.x (xx)	xx.x (95% CI)	P=	xx.x (xx)	xx.x (xx)	xx.x (95% CI)	P=	P=
LDL mmol/L	xx.x (xx)	xx.x (xx)	xx.x (95% CI)	P=	xx.x (xx)	xx.x (xx)	xx.x (95% CI)	P=	P=
HDL mmol/L	xx.x (xx)	xx.x (xx)	xx.x (95% CI)	P=	xx.x (xx)	xx.x (xx)	xx.x (95% CI)	P=	P=
Triglycerides mmol/L	xx.x (xx)	xx.x (xx)	xx.x (95% CI)	P=	xx.x (xx)	xx.x (xx)	xx.x (95% CI)	P=	P=
Blood Pressure (mm Hg) at 3, 6 mths									
Systolic BP	xx.x (xx)	xx.x (xx)	xx.x (95% CI)	P=	xx.x (xx)	xx.x (xx)	xx.x (95% CI)	P=	P=
Diastolic BP	xx.x (xx)	xx.x (xx)	xx.x (95% CI)	P=	xx.x (xx)	xx.x (xx)	xx.x (95% CI)	P=	P=
Weight (kg) at 3, 6mths	xx.x (xx)	xx.x (xx)	xx.x (95% CI)	P=	xx.x (xx)	xx.x (xx)	xx.x (95% CI)	P=	P=
BMI (weight (kg)/height (m^2) at 3, 6 mths	xx.x (xx)	xx.x (xx)	xx.x (95% CI)	P=	xx.x (xx)	xx.x (xx)	xx.x (95% CI)	P=	P=
Waist circumference (cm)	xx.x (xx)	xx.x (xx)	xx.x (95% CI)	P=	xx.x (xx)	xx.x (xx)	xx.x (95% CI)	P=	P=

	HN001				B-glucan				
	Yes	No			Yes	No			
	Mean (SD)	Mean (SD)	Mean diff (95% CI)	P=	Mean (SD)	Mean (SD)	Mean diff (95% CI)	P=	P for interaction
**Secondary outcomes: Mental health and wellbeing outcomes at 6 and 9 months**									
DASS Total score	xx.x (xx)	xx.x (xx)	xx.x (95% CI)	P=	xx.x (xx)	xx.x (xx)	xx.x (95% CI)	P=	P=
DASS Depression subscale	xx.x (xx)	xx.x (xx)	xx.x (95% CI)	P=	xx.x (xx)	xx.x (xx)	xx.x (95% CI)	P=	P=
DASS Anxiety subscale	xx.x (xx)	xx.x (xx)	xx.x (95% CI)	P=	xx.x (xx)	xx.x (xx)	xx.x (95% CI)	P=	P=
DASS Stress subscale	xx.x (xx)	xx.x (xx)	xx.x (95% CI)	P=	xx.x (xx)	xx.x (xx)	xx.x (95% CI)	P=	P=
SF 36	xx.x (xx)	xx.x (xx)	xx.x (95% CI)	P=	xx.x (xx)	xx.x (xx)	xx.x (95% CI)	P=	P=
Physical Functioning	xx.x (xx)	xx.x (xx)	xx.x (95% CI)	P=	xx.x (xx)	xx.x (xx)	xx.x (95% CI)	P=	P=
Role Physical	xx.x (xx)	xx.x (xx)	xx.x (95% CI)	P=	xx.x (xx)	xx.x (xx)	xx.x (95% CI)	P=	P=
Bodily Pain	xx.x (xx)	xx.x (xx)	xx.x (95% CI)	P=	xx.x (xx)	xx.x (xx)	xx.x (95% CI)	P=	P=
General Health	xx.x (xx)	xx.x (xx)	xx.x (95% CI)	P=	xx.x (xx)	xx.x (xx)	xx.x (95% CI)	P=	P=
Vitality	xx.x (xx)	xx.x (xx)	xx.x (95% CI)	P=	xx.x (xx)	xx.x (xx)	xx.x (95% CI)	P=	P=
Social Functioning	xx.x (xx)	xx.x (xx)	xx.x (95% CI)	P=	xx.x (xx)	xx.x (xx)	xx.x (95% CI)	P=	P=
Role Emotional	xx.x (xx)	xx.x (xx)	xx.x (95% CI)	P=	xx.x (xx)	xx.x (xx)	xx.x (95% CI)	P=	P=
Mental Health	xx.x (xx)	xx.x (xx)	xx.x (95% CI)	P=	xx.x (xx)	xx.x (xx)	xx.x (95% CI)	P=	P=
Physical Component Summary PCS	xx.x (xx)	xx.x (xx)	xx.x (95% CI)	P=	xx.x (xx)	xx.x (xx)	xx.x (95% CI)	P=	P=
Mental Component Summary (MCS)	xx.x (xx)	xx.x (xx)	xx.x (95% CI)	P=	xx.x (xx)	xx.x (xx)	xx.x (95% CI)	P=	P=

[a] Adjusted for time, baseline values of the outcome variable, plus baseline co-variates listed in Tables 1,2 and 3.

Supplement use, physical activity, diet, smoking, alcohol consumption and recreational drug use will also be collected at time 3, 6 and 9 months and tested for their interaction effect with the main exposures (HN001 and Beta-glucan) on the primary and secondary outcomes.

If interactions are significant ($p<0.05$), findings will be stratified by the interaction variable, and presented as below.

Table 5: The effects of HN001 and B-glucan and their interaction on the primary and secondary outcome variables stratified by ***interaction variable***

	**HN001**				**B-glucan**				
	Yes	No		P=	Yes	No		P=	P for interaction
	Mean (SD)	Mean (SD)	Mean diff (95% CI)	P=	Mean (SD)	Mean (SD)	Mean diff (95% CI)	P=	P=
**Interaction variable level 1**									
HbA1c(mmol/mol) at 6 mths (%)	xx.x (xx)	xx.x (xx)	xx.x (95% CI)	P=	xx.x (xx)	xx.x (xx)	xx.x (95% CI)	P=	P=
**Interaction variable level 2**									
HbA1c(mmol/mol) at 6 mths %	xx.x (xx)	xx.x (xx)	xx.x (95% CI)	P=	xx.x (xx)	xx.x (xx)	xx.x (95% CI)	P=	P=

Table 6: The effects of HN001 and B-glucan and their interaction on gastrointestinal adverse event prevalence.

	HN001				B-glucan				
	Yes	No			Yes	No			
	N (%)	N (%)	RR (95% CI)	P=	N (%)	N (%)	RR (95% CI)	P=	P for interaction
Increased nausea	n (%)	n (%)	RR (95% CI)		n (%)	n (%)	RR (95% CI)		
Increased stomach ache/cramps	n (%)	n (%)	RR (95% CI)		n (%)	n (%)	RR (95% CI)		
Increased bloated/swollen stomach	n (%)	n (%)	RR (95% CI)		n (%)	n (%)	RR (95% CI)		
Negative impact on bowel function	n (%)	n (%)	RR (95% CI)		n (%)	n (%)	RR (95% CI)		
