# Supplementary material for: Food 4 Health - He Oranga Kai: Assessing the efficacy, acceptability and economic implications of Lactobacillus rhamnosus HN001 and β-glucan to improve glycated haemoglobin, metabolic health, and general well-being in adults with pre-diabetes: study protocol for a 2 × 2 factorial design, parallel group, placebo-controlled randomized controlled trial, with embedded qualitative study and economic analysis
Source: Trials. 2019 Jul 29;20:464. doi: 10.1186/s13063-019-3553-7 (PMC6664750; doi:10.1186/s13063-019-3553-7)
Supplement: Supplementary file 5 — Food 4 Health - He Oranga Kai: Blood and faecal sample collection and quality control protocols. (PDF 705 kb) [file 13063_2019_3553_MOESM5_ESM.pdf]

Additional File 5

Food 4 Health - He Orangi Kai: Blood & faecal sample collection and quality control protocols

Blood collection and storage protocols

Fasting blood samples are collected at each study time point, then centrifuged, and plasma or serum placed in aliquots and frozen at -80 °C. See table Table 1 for further details. Quality control data collected for each sample include date and time collected, and date and time frozen. Any obvious quality issue such as visible haemolysis or lipaemia is noted.

Table 1: Blood sample collection, processing and proposed analysis

**Description**	**Becton Dickinson (BD) vacutainer code**	**Number Required**	**Draw order**	**After collection condition**	**Centrifuge instructions ***	**Samples for:**
**Vacutainers required for those consenting to bloods for Food 4 Health study only**						
K_2EDTA 4 ml	367839	1	1	ice slurry	3500 for 15 mins	HbA1c<br>Insulin<br>hs CRP
SST II Advance (yellow top)	367958	1	2	ambient Stand 30 mins	Check clot has formed before centrifuge 3500 for 15 mins	Fasting lipids
Grey top (fluoride oxalate) 2ml	367934	1	3	ice slurry	3500 for 15 mins	Fasting glucose
**Vacutainers required for those consenting to bloods for bio banking**						
P800 2ml	366420	2	4	ambient	3500 for 15 mins	
K_2EDTA 10 ml	367525	2	5	ice slurry	3500 for 15 mins	

*samples checked visually for clarity after spinning, and spun for a further 5 minutes if plasma remains cloudy

Blood samples are drawn using Push Button Blood Collection Sets with pre-attached Holder 21 gauge (BD code 368657) and 23 gauge (BD code 368658)

Faecal sample collection and storage protocols:

All participants taking part in the study will be asked to collect three faecal samples prior to the enrolment and all outcome visits. These samples are optional and collected for bio banking to enable mechanistic studies if interventions are efficacious.

- Sample 1: Collected into Stool Nucleic Acid Collection and Preservation Tube supplied by Norgen Biotek Corp Cat# 45660. DNA is stable for 2 years, and RNA for 7 days at room temperature in these tubes. Tubes are frozen at -80 °C to extend the stability of RNA and DNA.

- Samples 2 & 3: Collected into standard clean hospital grade faecal sample tubes, and then frozen immediately at -20 °C within a larger container containing water surrounding the sample to ensure the cold chain is preserved when the sample is transferred to the study centre. Once received at the study centre the ice is removed and samples are stored at -80°C

Timing of collections:

Participants are requested to commence the food diary record 6 days before each study visit, and following this collect the faecal samples.

All faecal samples are collected at the same time and within 2-3 days prior to study visit. This timing is to ensure there is adequate time to freeze samples 2 and 3 immediately after collection, and to minimise the time before samples are stored at -80 °C.

Quality control data collected:

All samples:

- Was sample contaminated by urine?
- Was sample contaminated by toilet water?
- Date and time collected
- Date and time placed at -80 °C

Sample 1 only:

- Was an excess amount of sample collected? (Tubes have a pre specified fill line)
- Was the sample adequately mixed?

Samples 2 & 3 only:

- How long after collecting the sample, did participant place sample at -20°C?
