# Supplementary material for: Food 4 Health - He Oranga Kai: Assessing the efficacy, acceptability and economic implications of Lactobacillus rhamnosus HN001 and β-glucan to improve glycated haemoglobin, metabolic health, and general well-being in adults with pre-diabetes: study protocol for a 2 × 2 factorial design, parallel group, placebo-controlled randomized controlled trial, with embedded qualitative study and economic analysis
Source: Trials. 2019 Jul 29;20:464. doi: 10.1186/s13063-019-3553-7 (PMC6664750; doi:10.1186/s13063-019-3553-7)
Supplement: Supplementary file 6 — Food 4 Health - He Oranga Kai: Dissemination strategy. (PDF 612 kb) [file 13063_2019_3553_MOESM6_ESM.pdf]

Additional file 6:

Dissemination strategy for Food 4 Health - He Oranga Kai

Outcomes from this study will be widely disseminated, and the qualitative methods and economic analysis are specifically designed to enhance this process and if positive outcomes are shown, to facilitate the effective translation into clinical practice.

Participant and whanau/family will be provided with lay summaries of the study results.

Professional colleagues and organisations assisting with recruitment will be provided with summaries of research outcomes and offered presentations.

Wider networks of professional colleagues (Including general practitioners, specialists, nurses, dietitians and other healthcare providers) most commonly learn from published peer reviewed journal articles, conference presentations and regional professional-specific peer review groups. Therefore, all of these traditional dissemination strategies will be important. Because of the global relevance of this research, we will target high impact diabetes journals and international as well as national conferences, including the specialist organisation such as the scientific meeting, and general practitioner meetings. Publication of study outcomes will be accompanied by media releases.

Dissemination of the results to the relevant sectors of the public will occur using established relationships such as Diabetes New Zealand, the National Heart Foundation NZ, Mental Health Foundation NZ, Health Navigator and Health Promotion Agency. Effective dissemination to Māori and Pacific people will directly involve these communities, and may take the form of hui/public meetings. We have previously successfully used Pacific Radio with good effect, and would look to use these avenues again.

Feedback to the Ministry of Health National Diabetes Leadership group and other relevant Ministry of Health representatives will occur if policy changes are indicated to improve the health outcomes of New Zealanders. Similarly, should the interventions be proven to be effective discussions will be held the PHARMAC (the New Zealand drug purchasing agency) and MEDSAFE (New Zealand's Medicines and Medical Devices Safety Authority) to explore pathways to funded access.

On a national scale outcomes from this research need to be integrated with other evidence based interventions for pre-diabetes. While CVD screening is well established and identifies this population group, high level work needs to be undertaken at Ministry of Health, District Health Board and Primary Health Organisation levels to implement a structured programme for those with pre-diabetes(1,2). Such a programme would link with primary care, whanau based or public health approaches already in place (green prescriptions, healthy families initiatives etc.) and simultaneously address factors such as diverse cultural understandings, health literacy and barriers to self-management. Contemporary diabetes care depends on an inter-professional team and the research team reflects this disciplinary mix and has contacts with the disciplines involved in clinical practice.

References:

1. Glasgow RE, Lichtenstein E, Marcus AC. Why Don't We See More Translation of Health Promotion Research to Practice? Rethinking the Efficacy-to-Effectiveness Transition. Am J Public Health. 2003;93(8):1261–7.

2. Glasgow RE. Translating Research to Practice: Lessons learned, areas for improvement, and future directions. Diabetes Care. 2003;26(8):2451–6.
